# Recent Progress in Carbon Fiber Reinforced Polymers Recycling: A Review of Recycling Methods and Reuse of Carbon Fibers

**DOI:** 10.3390/ma14216401

**Published:** 2021-10-25

**Authors:** José Antonio Butenegro, Mohsen Bahrami, Juana Abenojar, Miguel Ángel Martínez

**Affiliations:** 1 Materials Science and Engineering and Chemical Engineering Department, IAAB, University Carlos III Madrid, 28911 Leganés, Spain; mbahrami@ing.uc3m.es (M.B.); abenojar@ing.uc3m.es (J.A.); mamc@ing.uc3m.es (M.Á.M.); 2 Mechanical Engineering Department, Universidad Pontificia Comillas, Alberto Aguilera 25, 28015 Madrid, Spain

**Keywords:** polymer matrix composites, carbon fibers, recycling methods, circular economy

## Abstract

The rapid increase in the application of carbon fiber reinforced polymer (CFRP) composite materials represents a challenge to waste recycling. The circular economy approach coupled with the possibility of recovering carbon fibers from CFRP waste with similar properties to virgin carbon fibers at a much lower cost and with lower energy consumption motivate the study of CFRP recycling. Mechanical recycling methods allow the obtention of chopped composite materials, while both thermal and chemical recycling methods aim towards recovering carbon fibers. This review examines the three main recycling methods, their processes, and particularities, as well as the reuse of recycled carbon fibers in the manufacture of new composite materials.

## 1. Introduction

The demand for carbon fiber reinforced polymer (CFRP) composite materials is increasing rapidly, powered by a wide variety of industries such as general transport, including aerospace, automotive and sea vehicles; defense; wind turbines; construction; marine; sports; leisure; and storage tanks [1,2,3,4,5,6,7,8]. The growing demand for CFRP composites (four times the annual growth rate of global gross domestic product (GDP) per capita over the past decade [9,10,11]) in industry as a high-performance, light-weight materials is a result of their high specific strength, high specific stiffness, high fatigue resistance, good corrosion resistance, high durability, and low density. This last property is essential for aerospace and automotive industries, which strive to achieve energy efficiency while reducing the dependence on oil [12].

Fiber reinforced polymer (FRP) composite materials consist of a polymeric matrix and reinforcement fibers, making CFRP a subgroup of FRP. The matrix is usually a thermoset polymer, due to its better mechanical properties and better fiber-matrix adhesion, compared to thermoplastic polymers, but thermoplastics can be used as matrices as well. The most common polymers used as matrices are polyester, acrylic and epoxy resins [13,14]. Fibers enhance the mechanical properties of the composite material. Glass fibers have typically been the most economical choice among fibers. However, carbon fibers are employed for high-value, high-performance applications, where high specific properties are critical. Polyacrylonitrile (PAN) carbon fiber is the predominant type of fiber due to its relative ease of production and ability to maintain excellent mechanical properties. Other fibers, such as aramid, boron or basalt, are also being used for very specific applications, but their volumes are irrelevant compared to glass fibers or carbon fibers reinforced polymers [15,16,17,18]. Natural fibers are of interest since they can be extracted from renewable sources of animals, vegetable plants, and also minerals [19].

**Figure 1:**
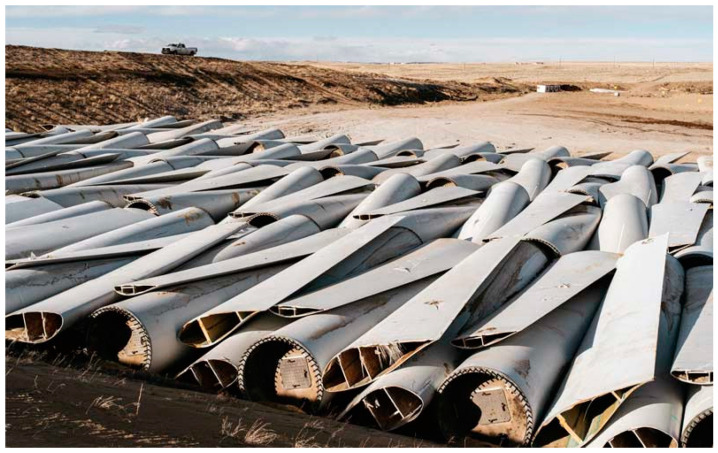
Wind turbine blades in a landfill (adapted from [22]).

However, the most important disadvantage of FRPs is the difficulty of recycling them. Landfilling and incineration have been the predominant recycling methods for a long time. However, these are not sustainable approaches since they are unable to solve the issues related to waste accumulation (landfilling) or require intensive energy consumption (incineration) [20]. FRP recycling is not carried out for economic reasons, as the recycled fibers obtained are short (therefore losing the added value of long fibers) and more expensive than virgin fibers. Thus, the motivation for recycling is not to reduce raw material costs but to cope with the high volume of FRP waste that is expected in the upcoming years, when wind energy elements and aircrafts reach their end-of-life [21]. An example of the environmental impact caused by the accumulation of FRP at end-of-life is shown in Figure 1.

The recycling issue has been addressed in Europe through a range of policies for several years now [23,24]. Current legislation on the recycling and reuse of composite materials is not very concise. In the case of Europe, in accordance with the Paris Agreement [25], the European Commission intends to reduce greenhouse gas emissions by at least 55% by 2030 and by at least 80% by 2050, both when compared to 1990 levels [26]. Regarding CFRP use and recycling in the automotive industry in the European Union, Directive 2000/53/EC establishes minimum reuse and recovery values of 95% for all end-of-life vehicles and reuse and recycling values of 85% by an average weight per vehicle per year. [27,28]. In order to achieve the proposed sustainability objectives, the industry must address three key points: the processes—improving them to reduce production time; the design—improving the distribution of loads in aerofoils for example; and the development of new materials—employing natural fibers or bio-based polymers [19,29].

Composite materials recycling is a complex issue due to multiple aspects. The use phase of composite materials dominates the life cycle energy consumption, especially for those applications that require an energy input, benefitting from these high-performance materials in terms of reduced consumption and emissions due to weight reduction [30,31,32]. Such applications include the aerospace industry or the manufacturing of pressure vessels, with an increasing presence of the automotive industry [33,34,35]. In such applications, the technical requirements are extremely demanding, which has historically justified the lack of incentives for recycling and recovering the waste generated, focusing on performance almost exclusively. Strategies for approaching the reduction of CFRP waste generation include, but are not limited to, prevention, minimization, reuse, recycling, energy recovery, and waste disposal, in descending order of priority.

Regarding the use of recycled carbon fiber (rCF), some of the problems that may arise as a result of the different recycling processes may include loss of strength [20,36,37], fiber damage [38,39], variation in fiber length [40,41,42,43], changes in fiber diameter [38,44], char deposition [45,46,47], and contamination on fibers [47,48,49]. From an environmental point of view, some recycling methods present significant problems of gas emissions [50] or the use of potentially hazardous solvents [42,51].

**Figure 2:**
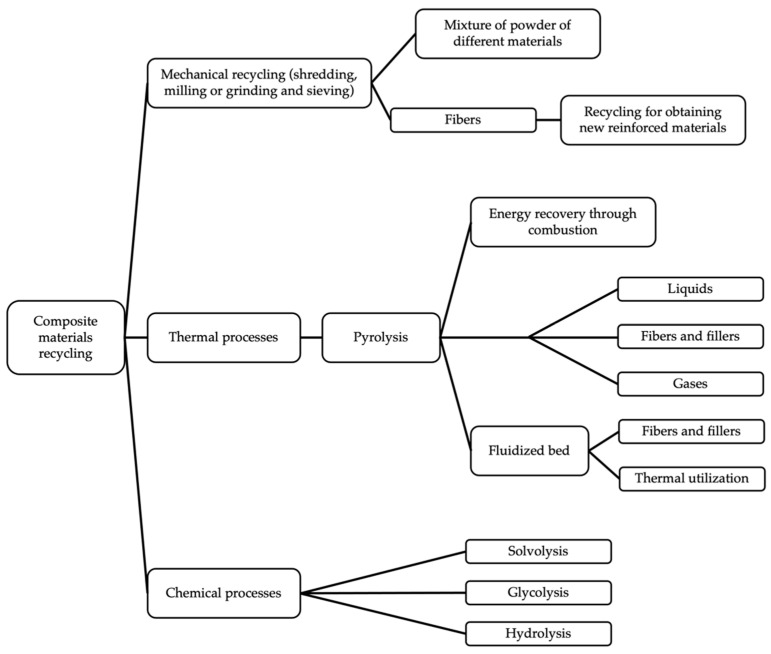
Composite materials recycling alternatives.

To approach the problems mentioned above, the issue of CFRP recycling has been approached from different points of view. There are three main recycling methods: mechanical, thermal, and chemical recycling. Mechanical recycling obtains chopped composite materials, while both thermal and chemical recycling aim to recover fibers. Landfilling and incineration, considered as recycling methods, are not sustainable and should be replaced as soon as possible. Pyrolysis is the most commonly used thermal method, allowing to obtain fibers with good mechanical performance if the process is optimized, and obtaining fillers and hydrocarbon liquids and solids as subproducts. Other subprocesses, such as fluidized bed are well established and documented. Chemical recycling consists of the recovery of fibers by degradation of the polymer matrix. Possible routes for degradation include solvolysis, hydrolysis, and glycolysis. These recycling approaches, as well as main techniques, subprocesses, and products obtained, can be observed in Figure 2.

## 2. Mechanical Recycling

Mechanical recycling consists of grinding, crushing, grounding, milling, and/or shredding the composite material and using it as a reinforcement with a new matrix. After grinding, sieving is usually carried out to obtain both powdery (resin-rich) and fibrous (fiber-rich) products. Unlike thermal and chemical recycling, mechanical recycling does not generate toxic gases such as carbon monoxide or greenhouse gases such as carbon dioxide. Some of the methods researched in the last years are collected in Table 1.

**Figure 3:**
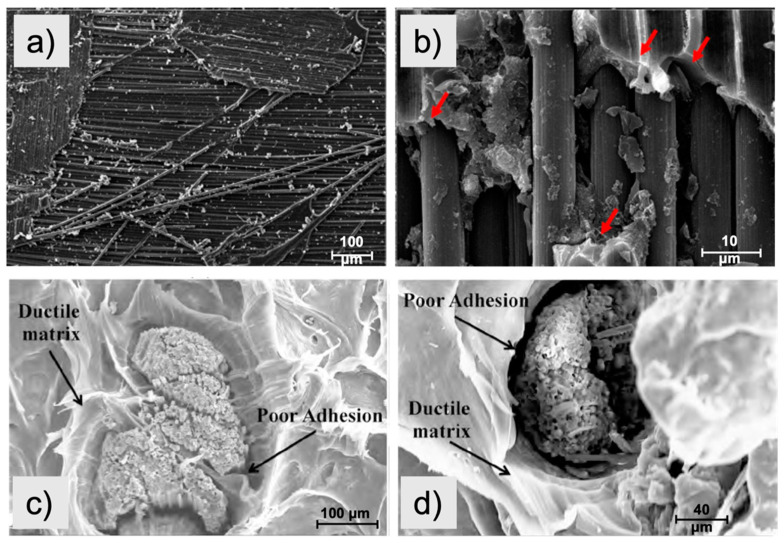
SEM images of the recycled carbon fibers: (**a**,**b**) poor epoxy-rCF interface [60]; (**c**,**d**) poor adhesion between the PP matrix and rCF [61].

The main problem with mechanical recycling is the adhesion between the recycled material and the new matrix. Figure 3 exhibits some examples of this issue in the literature. Figure 3a shows a SEM image for epoxy-rCF interface. In Figure 3b, with higher magnification, arrows exhibit complete separation between the epoxy matrix and rCF. Similarly, Figure 3c,d displays the fracture surface images of the polypropylene (PP) composite and poor adhesion between rCF and the matrix due to the agglomerated fibers in the polymeric matrix.

Okayasu et al. [54] found that when material made from ABS-matrix recycled material was subjected to tensile stresses, failure occurred mainly by pull-out of the fibers in the matrix. Palmer et al. [55] optimized the process of mixing virgin carbon fiber (vCF) and mechanically recycled carbon fiber and manufacturing composites by DMC, reducing the cost of these manufactured composites by minimizing the ratio of vCF to rCF, while maintaining mechanical properties.

Due to typically damaging fibers and reducing fiber length, mechanical recycling is used as a pre-recycling process for thermal or chemical recycling [20]. The reduction in the mechanical performance of mechanically recycled composites can be explained mainly because the carbon fibers are discontinuous; therefore, recycled composite properties are not comparable to those of long carbon fiber or continuous carbon fiber composites. Chen [62] illustrates the drop in mechanical properties due to shredding, showing a reduction in tensile strength and flexural strength of about 65% and 85%, respectively. The reduction in modulus of elasticity (MOE) is less pronounced, with a drop in tensile MOE and flexural MOE of 50%.

As an alternative to shredding, Roux et al. [58] used the electrodynamic fragmentation method to reduce door hinges from the aerospace industry. By controlling the applied voltage, they were able to reduce the composite material without mechanical shredding, resulting in a decrease of only 17% of the mechanical performance compared to the original product. Nonetheless, a continuous flow working machine would be required to implement this process in an industrial application.

## 3. Thermal Recycling

**Figure 4:**
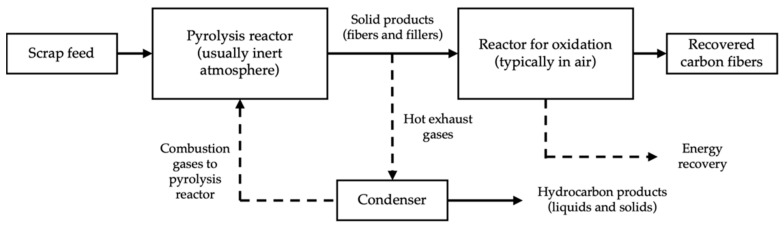
Pyrolysis process (modified from Pickering et al. [63]).

Thermal recycling methods focus on the recovery of the carbon fiber by breaking down the matrix. Pyrolysis and oxidation in fluidized bed are the two most used methods in thermal recycling. Energy recovery is discarded as a recycling method to recover carbon fibers, as all the added value of long carbon fibers compared to short fibers in CFRP manufacturing is lost. A simplified model for pyrolysis is represented in Figure 4.

### 3.1. Pyrolysis

In pyrolysis, the CFRP is heated up to the range of 350 to 700 °C in an inert atmosphere. The composite material decomposes and produces gases, bio-oil and solids (fibers, fillers and carbonaceous residue (char)). The mechanical performance of recovered carbon fibers is highly dependent on the parameters of the process. In this regard, the temperature for the process must be carefully selected attending to the matrix. If this temperature is too low, char remains adhered to the fibers, while temperatures too high produce a decrease in the thickness of fibers. A post-pyrolysis treatment, consisting of oxidation with air, is required to remove the solid carbon contamination, obtaining clean fibers and fillers [64]. This stage may result in a significant loss in the tensile strength of the fibers.

The two main advantages of pyrolysis are the ability to obtain rCF that retain at least 50 to 75% of the mechanical properties (or a 90–95% of the mechanical properties if pyrolysis is followed by an oxidation and both processes are optimized) and the possibility to be implemented at a commercial scale. On the downside, pyrolysis requires an inert atmosphere and a complementary oxidation process to improve the mechanical properties of the fibers obtained. In addition, the environmental impact of this process, which usually involves maintaining temperatures above 500 °C and emitting hazardous gases, poses a sustainability problem. Several thermal recycling methods, focusing on pyrolysis, are presented in Table 2.

Nahil et al. [47] proved that by performing pyrolysis at 500 °C, followed by oxidation at 500 °C, it is possible to maintain more than 90% of the tensile strength and elastic modulus (compared to virgin fibers). Abdou et al. [74] used a TGA for carbon fiber reinforced epoxy, at 550 °C for 1 h in a nitrogen atmosphere, to recover carbon fibers with no pores, fracture or carbonization.

Microwave-assisted pyrolysis (MAP) processes consist of heating the material from the inside by means of microwaves, avoiding char formation [75]. The energy consumption of MAP, compared to conventional pyrolysis, tends to be much lower. Hao et al. [76] successfully recovered carbon fibers from prepreg under microwave pyrolysis, followed by oxidation. They observed a strength reduction lower than 20% compared to the original prepregs, which could be further diminished by reducing the pyrolysis temperature.

Wu et al. [73] studied the catalytic pyrolysis of carbon fiber reinforced epoxy in molten ZnCl_2_. They found that the molten salt prevented oxidation during pyrolysis, that the temperature required for pyrolysis was significantly low (380 °C), and that the tensile strength of the recovered fibers was near 95% of the tensile strength of virgin fibers. Despite flexural properties and interlaminar shear strength (ILSS) showing some decrease compared to the composites made with vCF, after a sizing treatment, the properties of these composites were again very similar to those of vCF composites.

### 3.2. Fluidized Bed

Fluidized bed has been developed for the latest 20 years and operates at a pilot-scale. In these processes, the CFRP waste is fluidized typically under pressure (10–25 kPa) by a hot air stream (450–550 °C) in a silica sand bed. The high temperature decomposes the matrix without damaging the fibers, which can be separated and recovered, these being oxidized at a later stage with the gases released by the matrix. Some of the risks involved in this technique are the presence of organic solvents and the emission of polluting gases. Besides the high economic cost of maintaining a continuous stream of hot air, the major drawback of the fluidized bed process is the severe decrease in the mechanical properties of the fibers during recovery.

Pickering et al. [37] built a commercial-scale fluidized bed plant, which resulted in an energy decrease required to obtain rCF from 90 to 95% compared to vCF. The fibers obtained only suffered an 18.2% loss of their tensile strength while maintaining intact their tensile modulus.

## 4. Chemical Recycling

Chemical recycling, along with thermal recycling, is the method in which most research effort is currently being invested. The motivation behind the development of chemical recycling is based on achieving rCF with unaltered mechanical properties or morphology after recycling, as well as reducing the high energy consumption of thermal recycling [77]. The best mechanical performance for rCF is usually achieved by chemical recycling methods. Some of the factors that influence the outcome of the process are temperature, pressure, catalysts, and solvents used. The temperatures used in the different techniques that can be grouped into chemical recycling are generally lower than 400 °C. Solvolysis processes can be divided according to their requirements: higher temperature (>200 °C) and high pressure or lower temperature (<200 °C) and low pressure [78].

The degradation of the resin can be achieved by means of water (hydrolysis) or solvents (solvolysis). To minimize the environmental problems as well as the harmfulness and toxicity of hazardous and concentrated chemicals, much research is focused on the use of subcritical or supercritical water and alcohol as a substitute for these chemicals. Some possibilities for matrix decomposition include depolymerization or alkaline digestion, in which benzyl alcohol and tripotassium phosphate are used; acid digestion, using acetic acid and hydrogen peroxide to remove the epoxy resin; and sub- (low pressure and temperature) and supercritical (high pressure and temperature) fluids, employing water, ethanol, methanol, propanol or potassium hydroxide [49,79,80]. Besides the hazardous nature of the products used, the main disadvantage of chemical recycling is the very high difficulty of bringing these processes to a commercial scale. Diverse chemical methods studied in the last years are included in Table 3.

Sun et al. [46] proposed an electrochemical method for carbon fiber recycling from CFRP as a simple, effective, and economical recycling method. By identifying the parameters that affect recycling efficiency, they determined that an increase in electrolyte concentration does not yield better results in terms of efficiency. Moreover, the surface chemistry showed a loss of rCF crystallinity compared to that of vCF. The recycling rate, understood as the ratio of recycled depth to time, is extremely low.

Several authors have investigated the use of supercritical solvents, particularly acetone. One of the drawbacks of using sub- or supercritical solvents is the energy required to bring them to that state, either by pressure, temperature, or a combination of both. Okajima et al. [90] employed supercritical methanol, 1-propanol, 2-propanol, 1- butanol, 2-butanol, tert-butanol, acetone, and methyl ethyl ketone. While the results depend on the solvent, sub- and supercritical acetone were selected as optimal for rapidly degrading the matrix. Carbon fibers recovered with supercritical acetone maintain the tensile strength, and no matrix residues are observed on the fibers. Sokoli et al. [92] used supercritical acetone to recover glass fibers and carbon fibers from FRPs. While the recovered glass fibers had matrix residues in the fibers, the carbon fibers were recovered perfectly clean, with their mechanical properties intact. The authors point out the possibility of upscaling solvolysis processes, as all product streams are potentially reusable.

Das et al. [48] investigated the use of peracetic acid, formed from a mixture of acetic acid and hydrogen peroxide, as an oxidative method to recover carbon fibers from CFRP composites. The surface of rCF is clean, and the tensile strength is comparable to that of vCF. In addition, the solvents are recovered in pure and reusable form with a recovery efficiency above 90%. Coupled with the fact that the method does not require high temperatures and pressures, it results in a lower environmental impact.

Khalil [95] compared seventeen supercritical fluids commonly used for depolymerizing thermoset resins in CFRP waste, providing numerical examples to demonstrate that higher reaction temperature and pressure in solvolysis leads to higher resin removal efficiency but also leads to a much bigger environmental footprint. The study ranked supercritical fluids in terms of cradle-to-gate (C2G) production energy intensity, finding supercritical mixtures of solvents and water more effective in the recovery of carbon fibers but also requiring less production energy intensity, therefore causing lesser environmental footprint. Once again, the main disadvantage of chemical recycling is the very high difficulty of bringing these processes to a commercial scale.

## 5. Reuse of Recycled Carbon Fibers

**Figure 5:**
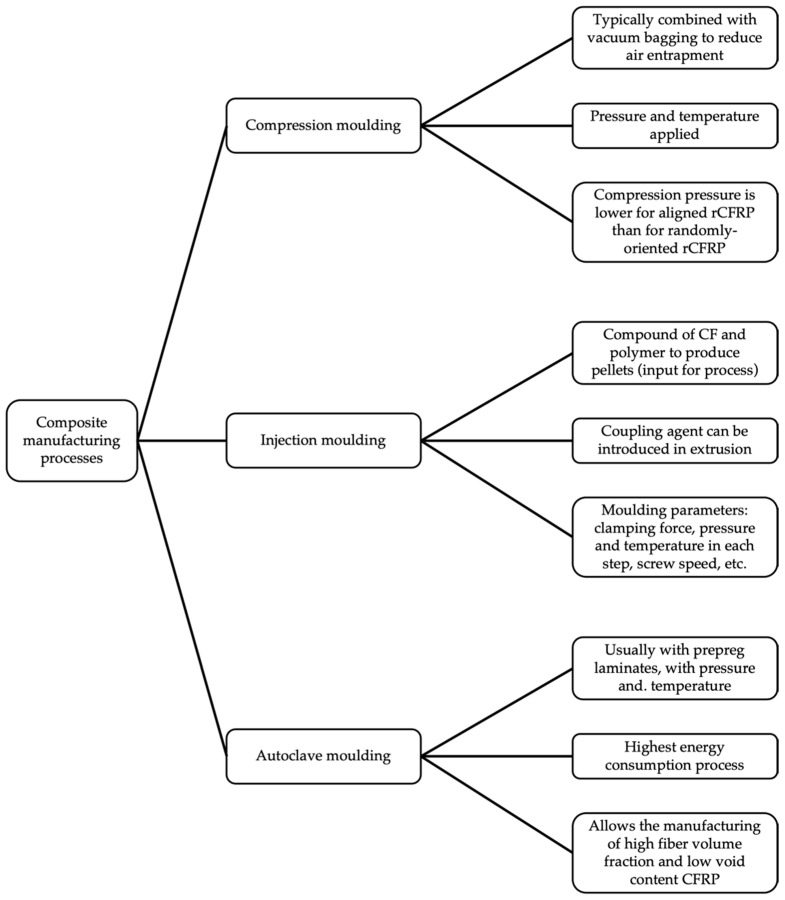
Most common composite manufacturing processes for rCFRP (adapted from Meng et al. [30]).

Aiming towards the objectives of a circular economy, carbon fiber recovery is a key factor. In terms of costs and energy, the recovery and reuse of rCF are perfectly justified. While producing vCF is an energy-intensive process, the energy cost of recycling carbon fibers can be 82–98% lower, therefore leading to a significant cost reduction [96]. Since the mechanical properties of rCF are not much lower than those of vCF, CFRP manufactured with rCF (rCFRP) can achieve even higher values of tensile strength or impact resistance compared to commercial CFRP. Those rCFRPs are typically manufactured by compression moulding, injection moulding, or autoclave moulding. These main composite manufacturing processes and their typical steps are reviewed in Figure 5.

The mechanical performance of rCFRP is deeply influenced by two factors: fiber length and wettability/adhesion between fiber and matrix. Carbon fiber length is affected by recycling processes, either by controlled cutting before the recycling (to recycle, the CFRP has to fit in an oven for pyrolysis, in a reactor, etc.) or after, or because carbon fibers break down during recycling. Matrices can be thermoset polymers or thermoplastic polymers.

As thermosets, epoxy and polyester have been the most used polymers. CFR thermoset polymers are usually manufactured by compression moulding or autoclave moulding. Sukanto et al. [97] enumerate three different automotive components manufactured with rCF: bulk moulded compounds (BMC) in the form of epoxy resin and calcium carbonate (CaCO_3_) mixture; a sheet moulded compound (SMC), filler-epoxy; and prepregs, rCF-epoxy resin. Gopalraj et al. [45] obtained rCFs and recycled glass fibers with a cone calorimeter setup, achieving a recovery rate of rCFs of 95–98% in weight. Those rCFs were then used to manufacture unidirectional CFRP with an epoxy resin, successfully achieving the closed-loop recycling. Mantelli et al. [98] successfully used 3D printing of rCFs. They shredded pyrolyzed rCF with a sizing treatment and used those shreds as a reinforcement of a thermally and photo-curable thermoset resin. The authors point out that a better fiber-matrix adhesion could be achieved by selecting a specifically designed sizing agent. Some examples of the use and reuse of recycled carbon fibers and recycled carbon fiber reinforced polymers are shown in Table 4.

Roux et al. [58] fragmented carbon fiber reinforced PEEK from an aerospace application (door hinges) via electrodynamical fragmentation and then manufactured new door hinges by compression moulding. The results show that this method improves mechanical performance compared to composites manufactured with rCF obtained by other mechanical methods, especially for aerospace applications. Huang et al. [106] recovered carbon fibers by means of a supercritical fluid and manufactured composites with an additive manufacturing-based approach. After recovering carbon fibers by supercritical fluid and grounding the rCFs, an extruder was used for obtaining rCF/PEEK composite filaments. For the fabrication, those filaments were then fed to a 3D printer.

As observed in the abovementioned examples, thermoplastic matrices can be used in industries such as aerospace or the automotive industry, normally for non-critical, non-structural parts. The application of thermoplastic polymers as matrices along with rCF or rCFRP leads to several advantages [107]. Thermoplastic matrices can benefit from the many advantages carbon fibers can offer in terms of mechanical, thermal, or electrical properties. Carbon fiber reinforced thermoplastic (CFRTP) composites can be recycled and reformed. This is a key insight since the amount of CFRTP to be recycled grows every day due to the increasing use in aerospace, automotive or chemical industries. As mentioned above, the cost of rCF is significantly lower than that of vCF, thus allowing potential entry to a greater number of markets.

On the other hand, carbon fibers have poor wettability and adhesion to thermoplastic matrices, therefore requiring surface treatments or the use of different substances [100,103]. The presence of residual solvent in the carbon fibers, used to decrease the processing cost, is a disadvantage that leads to a reduction in the performance of CFRTP.

## 6. Conclusions

In this report, current processes and methods for recycling composites have been reviewed, distinguishing between those aiming to obtain chopped composite materials and those whose objective is to recover carbon fibers. Mechanical recycling is not as attractive as it used to be as a process but still retains importance as a pre-treatment as part of thermal or chemical recycling methods. Thermal and chemical processes are nowadays receiving almost all the attention in terms of research and investment. Mechanical performance of recovered carbon fibers through thermal and chemical methods is typically higher than that of mechanically recovered carbon fibers.

One of the most essential issues with mechanical recycling is the adhesion between the recycled material and the new matrix. Since fiber length is usually reduced and fibers might suffer damage from mechanical recycling, much added value is lost by mechanical recycling. Other methods, such as electrodynamical fragmentation, are being studied more intensely.

Thermal recycling methods succeed in recovering carbon fibers by means of heat, being pyrolysis the most employed method. Carbon fibers recovered through pyrolysis in an inert atmosphere require an oxidation process afterwards to achieve optimal performance. Alternatives like MAP are also being developed. Fluidized bed processes allow the recovery of fibers in a very efficient way in terms of energy consumption, but the fibers recovered are fluffy. Energy recovery is discarded as a recycling method to recover carbon fibers, as all the added value of long carbon fibers compared to short fibers in CFRP manufacturing is lost.

The best mechanical performance for rCF is usually achieved by chemical recycling methods. The degradation of the resin is possible by hydrolysis, solvolysis and glycolysis. Due to the lower energy consumption compared to pyrolysis, much research is focused on reducing the environmental impact of chemical recycling. In that regard, the use of subcritical or supercritical water and alcohol as a substitute for hazard and concentrated chemicals is being studied.

The reuse of rCF is critical for a circular economy approach. rCFs are cheaper to produce compared to vCFs, both in terms of cost and energy consumption. By selecting the most appropriate recycling method and a matrix suitable for the wanted application, rCFRPs can successfully be implemented in most industries for non-critical, non-structural applications. The manufacturing processes that can be used with rCFRP include, but are not limited to, wet lay-up, prepreg lamination, injection moulding, compression moulding, 3D printing or resin transfer moulding (RTM).

The most significant research gaps that have been identified in this report are highlighted below.

- Composite waste does not have a homogeneous composition, neither in terms of matrices nor in terms of reinforcements. Neither does it come in a homogeneous type, being able to find cured/partially cured prepregs, loose fibers and recycled composites or fibers. This makes adapting the process conditions and material a challenging task.
- The lack of adhesion between matrix and recycled fibers prevents a greater use of these. New approaches that improve fiber-matrix adhesion or modifications to the current processes are required to solve this critical issue.
- Processes such as electrodynamical fragmentation or microwave-assisted pyrolysis require further research before being optimized and made applicable on an industrial scale.
- Processes such as fluidized bed or solvolysis using solvents in critical conditions still require extensive research before being fully functional on a commercial scale.
- Economic and energy analysis of thermal and chemical processes should be studied in more depth. Simulation models can be developed and used to include all phases in cradle-to-cradle life cycle assessment and to obtain a more accurate cost assessment against which to compare viable processes.

To summarize, CFRP recycling faces three key issues. First, the reduction in fiber length as a result of recycling processes reduces the performance of rCF compared to vCF. Then, energy recovery as a recycling method not only fails to recover the energy invested in the production of the composite material but also results in the total loss of the added value that the long carbon fibers provide. Finally, the adhesion between the new matrix and the rCFs is not ideal, which leads to the sub-optimal behavior of composites made with rCFs. With the demand for CFRP increasing every year and due to the significant environmental impact of these materials at the end of life, research is progressing to overcome these issues.

## Figures and Tables

**Table 1:** Mechanical recycling methods.

Material	Grinding Equipment	Size	Contribution	Ref.
GFR ^1^ polyester, CFR ^2^ epoxy and epoxy-based aramid fibers composites	Mini granulator + sieves from 1 to 5.5 mm	4–9 mm length, 8–12 μm diameter. (l/d_exp_ > l/d_crit_)	Introduction of mechanically recycled fibers in thermoplastic matrices (ethylene and methacrylic acid/propylene copolymer)	[52]
CFR epoxy	Rotating blade with a sieve/ball mill	1–10 mm 1–10 μm	ABS ^3^ matrix with recycled CFRP	[53,54]
GFR polyester (SMC ^4^)	Hammer mill. Sorting by air cascade	2–25 mm	Introduction of recycled fibers for DMC ^5^	[55]
CFR epoxy with different fiber types (woven, UD ^6^, ±45°). Several curing stages	Slow speed granulator + sieving	600 μm–11.2 mm	Study of granulator effectiveness	[56]
Commercial rCF (by depolymerization)	-	7 mm length	Enhancement of rCF-matrix adhesion with plasma treatments	[57]
CFR PEEK ^7^	Electronic equipment + sieving	2–10 mm length, 0.16–2 mm thickness	Electrodynamical fragmentation as an alternative to mechanical shredding	[58]
CFR epoxy	Microfine mill	20–100 μm	Absorption of PMMA ^8^ particles to improve adhesion with the new matrix	[59]

^1^ Glass fiber reinforced; ^2^ Carbon fiber reinforced; ^3^ Acrylonitrile butadiene styrene; ^4^ Sheet moulding compound; ^5^ Dough moulding compound; ^6^ Uni-directional; ^7^ Polyether ether ketone; ^8^ Poly(methyl methacrylate).

**Table 2:** Thermal recycling methods.

FRP	Reactor	Pyrolysis			Oxidation	Contribution	Ref.
		T (°C)	Time	Gas			
CFR epoxy	Py-GC/MS ^1^	700	–	–	–	Study of two composite materials	[65]
CFR epoxy	TGA ^2^	900	Variable	N_2_^3^	Air at 600 °C	Optimization of the pyrolysis cycle	[66]
CFR polybenzoxazine	Fixed-bed batch	350–700	1 h	N_2_	Air at 500 and 700 °C	Recovery of activated carbon fibers	[47]
CFR epoxy	Furnace	550	20 min 500 °C, 90 min 550 °C	N_2_	CO_2_^4^ + O_2_^5^ + air + H_2_O ^6^ at 550–700 °C	Chemical post-treatment in HNO_3_^7^	[67]
CFR epoxy cured, uncured and contaminated	Batch furnace, commercial process	<400	Sample dependent	–	–	PPS ^8^ material (thermoplastic) with rCF	[68]
CFR polybenzoxazine	Pilot-scale facility	500–700	–	–	Gasification in air at 500 °C	Process optimization	[69]
CFR epoxy	Fixed bed reactor	550	30 min	H_2_O	Air at 550 °C for 30 to 75 min	Carbon fiber recovery by super-heated steam method	[70]
Cured and uncured epoxy CFR cuts	Pilot plant batch	500	150 min	N_2_	Air at 500–600 °C for 10 to 60 min	Recovery of recycled fibers and production of new composites	[71]
CFR epoxy	TGA	500–1000	Variable	N_2_/ CO_2_	–	Use of CO_2_ and water vapor to remove char	[72]
CFR epoxy	Furnace	360	80 min	Air	–	Carbon fibers recovery through catalytic pyrolysis in molten ZnCl_2_^9^	[73]
CFR epoxy	Cone calorimeter (batch reactor)	550	20–25 min	–	–	Recovery of carbon fibers from discarded UD composites	[45]
CFR epoxy	TGA and furnace	300–700	60–120 min	N_2_	–	Process optimization	[74]

^1^ Pyrolysis-gas chromatography and mass spectrometry; ^2^ Thermogravimetric analysis; ^3^ Nitrogen; ^4^ Carbon dioxide; ^5^ Oxygen; ^6^ Water; ^7^ Nitric acid; ^8^ Polyphenylene sulphide; ^9^ Zinc chloride.

**Table 3:** Chemical recycling methods.

Chemical Agents	Reaction Conditions	Tensile Strength Retention	Ref.
Nearcritical and supercritical water	250–400 °C, 4–27 MPa, 1–30 min	90–98%	[81]
Water + benzyl alcohol	400 °C, 1 h	–	[79]
NaCl ^1^ dissolution	Electrochemical (4–25 mA)	80%	[46]
Hydrochloric acid in tetrahydrofuran	Room temp., 24 h	Similar to virgin fibers	[82]
AlCl_3_^2^ + Acetic acid	180 °C, 6 h	97.77%	[83]
Ethylene glycol	180 °C, 4 h	95%	[84]
Supercritical 1-propanol with 1% KOH ^3^	330 °C, 1 h	94.6%	[85]
Sub- and supercritical water and water/ethanol (50:50)	350–400 °C, 25 MPa	Similar to desized virgin fibers	[86]
ZnCl_2/_KOH/HPW ^4^/MgCl_2_^5^_/_AlCl_3/_FeCl_3_^6^ + ethanol/water	80–250 °C, 2–10 h	–	[87]
Water/acetone (20:80)	320 °C, 60 min	>90%	[88]
Supercritical n-butanol	360 °C, 1 h	98.63%	[89]
Supercritical acetone	320 °C, 20 min	Negligible decrease	[90]
Subcritical water Supercritical water	400 °C, 15 min 280 °C, 30 min	>90%	[91]
Peracetic acid (acetic acid + H_2_O_2_^7^)	65 °C, 4 h	Similar to virgin fibers	[48]
Nearcritical water and supercritical acetone	260–300 °C, 6–30 MPa	Similar to virgin fibers	[92]
Superheated and supercritical acetone	350 °C, 2–14 MPa, 60 min	–	[93]
Benzyltrimethylammonium bromide (BTAB) and sodium dodecyl sulfate (SDS)	Process: 100 °C, 1 hDry: 100 °C, 24 h	96.9%	[94]

^1^ Sodium chloride; ^2^ Aluminium chloride; ^3^ Potassium hydroxide; ^4^ Phosphotungstic acid; ^5^ Magnesium chloride; ^6^ Ferric chloride; ^7^ Hydrogen peroxide.

**Table 4:** General characteristics, manufacturing conditions, and mechanical properties in the reuse of recycled CF and CFRP.

Feedstock	Recycling Process	New Matrix Material	Conditions for Manufacturing	Mechanical Properties (rCF or Composite)	Ref.
CFR epoxy	Crushing	ABS/PP ^1^	Pelletizing of CFRTP (CFRP + thermoplastic polymer) using a two-axis pelletizing machine, followed by injection moulding	Composite: 24% fiber volume fraction seems to be a limit for mechanical properties	[99]
CFR epoxy	Mechanical cutting (chopping)	Epoxy	Fibers are converted to non-woven mats by a wet papermaking process. Compression moulding at 7 MPa	rCF: 98.1% TS ^1^, 95.6% TM ^2^ compared to virgin fibers	[38]
CFR epoxy and CFR bismaleimide	Mechanical cutting, pyrolysis at 400 °C, cleaning with water washing, including ultrasonication + fiber drying	PPS	Pelletizing using twin-screw extruder: throughput 3 kg/h, screw speed 150 rpm, die temperature 315 °C and cooling in air. Manufacturing by moulding press at 290–305 °C	Composite: 680% TM, 720% TS, 250% impact energy increase, when compared to PPS	[68]
CFR epoxy	Total cure of prepregs for 5 h at 100 °C. Mechanical shredding and sieving. Fluidized bed at 550 °C, followed by oxidation at 850 °C	PP	Compounding: using twin-screw extruder, L/D ratio 25:1, screw speed 50 (lower fiber damage)–80 rpm, coupling agents (2–8%) Injection moulding: nozzle temperature 200–210 °C, mould temperature 50 °C, hold pressure 12 MPa, back pressure 47.5 MPa	Composite: 150% TS increase when incorporating coupling agents (5% wt.) compared to composite with no coupling agents	[100]
rCF from CFR epoxy	As-received: Pyrolysis at 500 °C for 10 min, and cut	PP	Carding and wrap spinning process. Hot compression moulding, at 220 °C, 2 MPa for 15 min	rCF: retains 90% TS and 93% TM compared to vCF Composite: rCFRP with 27.7% volume fraction has 50% higher TS and FS ^3^ compared to rCFRP with 15% volume fraction	[101]
CFR epoxy	Crushed using rotating blade, ball milling process	ABS	Mixing, grinding and injection	Composite: Higher TS when higher content in CF, but drops dramatically at 70% (in weight)	[54]
CFR epoxy	Mechanical cutting, pyrolysis at 500 °C followed by oxidation at 500/600 °C	Epoxy	rCF chopping, oxidation (optional), mixing with epoxy resin and hot pressing at 110 °C, 4.5 MPa, for 40 min	rCF: 65–95% TS when compared to vCF	[102]
CFR epoxy	Solvolysis in water and acetone (20:80 in volume)	Epoxy	For rCF: manually alignment, impregnation with resin, and vacuum in bag. Cured at room temperature for 16 h and post-cured at 75–80 °C for 1 h For rCF plies: alignment, impregnation with resin, followed by curing in hot press at 60 °C, 3.5 MPa, for 5 h	rCF: >90% TS, compared to vCF	[88]
CFR polybenzoxazine	Mechanical crushing, followed by pyrolysis at 500 °C	LDPE ^4^	For blending, roll mill: speed 10–20 rpm, roll temperature 150–180 °C. Grounded and hot pressed at 34.5 MPa at 180 °C	rCF: Some combinations of rCF + additives show similar properties to vCF composites	[103]
CFR PEEK	Electrodynamical fragmentation, 6 cycles of 100 pulses, 180 kV, frequency 5 Hz, followed by sieving	PEEK	Compression moulding, 20 ton clamping force, heated at 360 °C for 3 min, cooled at a rate of 20 °C/min	Composite: rCFRP mechanical performance is 17% lower than novel composite	[58]
PAN-rCF	Unknown	PC ^5^	Pelletizing using twin-screw extruder: screw speed 100 rpm, die temperature 230–250 °C. Injection (biocarbon fillers + rCF) at 80–120 MPa, at 250 °C	Composite: 35% TM 270% TS increase, compared to the reference PC-biocarbon composite	[104]
CFR epoxy	Pyrolysis in molten ZnCl_2_ at 360 °C	Epoxy	Manual lay-up, cured at 80 °C for 2 h and at 150 °C for 4 h in oven.	rCF: 95% TS retention after pyrolysis in molten ZnCl_2_, 80% TS retention after pyrolysis in air, compared to vCF	[73]
rCF	Pyrolysis. Two treatments for resizing: Acetone washing and drying in oven Acidic treatment in a bath of 65% HNO_3_ for 20 min at 60 °C, followed by drying in oven	PP/PA6 ^6^	Preparation of films with 1–5% (in weight) of solids content. Drying for 12 h at 80 °C. Chopping of fibers. Compounding in a twin-screw microcompounder at 80 rpm, 190 °C for PP, 230 °C for PA6, compounding time 1 min	Composite: No effect of PP sizing, slight positive effect of PU ^7^ sizing on TS and TM	[105]

^1^ Tensile strength; ^2^ Tensile modulus; ^3^ Flexural strength; ^4^ Low density polyethylene; ^5^ Polycarbonate; ^6^ Polyamide 6; ^7^ Polyurethane.

## Data Availability

No new data were created or analyzed in this study. Data sharing is not applicable to this article.
